# Time trends and prescribing patterns of opioid drugs in UK primary care patients with non-cancer pain: A retrospective cohort study

**DOI:** 10.1371/journal.pmed.1003270

**Published:** 2020-10-15

**Authors:** Meghna Jani, Belay Birlie Yimer, Therese Sheppard, Mark Lunt, William G. Dixon

**Affiliations:** 1 Centre for Epidemiology Versus Arthritis, Centre for Musculoskeletal Research, University of Manchester, Manchester, United Kingdom; 2 Department of Rheumatology, Salford Royal NHS Foundation Trust, Salford, United Kingdom; Massachusetts General Hospital, UNITED STATES

## Abstract

**Background:**

The US opioid epidemic has led to similar concerns about prescribed opioids in the UK. In new users, initiation of or escalation to more potent and high dose opioids may contribute to long-term use. Additionally, physician prescribing behaviour has been described as a key driver of rising opioid prescriptions and long-term opioid use. No studies to our knowledge have investigated the extent to which regions, practices, and prescribers vary in opioid prescribing whilst accounting for case mix. This study sought to (i) describe prescribing trends between 2006 and 2017, (ii) evaluate the transition of opioid dose and potency in the first 2 years from initial prescription, (iii) quantify and identify risk factors for long-term opioid use, and (iv) quantify the variation of long-term use attributed to region, practice, and prescriber, accounting for case mix and chance variation.

**Methods and findings:**

A retrospective cohort study using UK primary care electronic health records from the Clinical Practice Research Datalink was performed. Adult patients without cancer with a new prescription of an opioid were included; 1,968,742 new users of opioids were identified. Mean age was 51 ± 19 years, and 57% were female. Codeine was the most commonly prescribed opioid, with use increasing 5-fold from 2006 to 2017, reaching 2,456 prescriptions/10,000 people/year. Morphine, buprenorphine, and oxycodone prescribing rates continued to rise steadily throughout the study period. Of those who started on high dose (120–199 morphine milligram equivalents [MME]/day) or very high dose opioids (≥200 MME/day), 10.3% and 18.7% remained in the same MME/day category or higher at 2 years, respectively. Following opioid initiation, 14.6% became long-term opioid users in the first year. In the fully adjusted model, the following were associated with the highest adjusted odds ratios (aORs) for long-term use: older age (≥75 years, aOR 4.59, 95% CI 4.48–4.70, *p <* 0.001; 65–74 years, aOR 3.77, 95% CI 3.68–3.85, *p <* 0.001, compared to <35 years), social deprivation (Townsend score quintile 5/most deprived, aOR 1.56, 95% CI 1.52–1.59, *p <* 0.001, compared to quintile 1/least deprived), fibromyalgia (aOR 1.81, 95% CI 1.49–2.19, *p <* 0.001), substance abuse (aOR 1.72, 95% CI 1.65–1.79, *p <* 0.001), suicide/self-harm (aOR 1.56, 95% CI 1.52–1.61, *p <* 0.001), rheumatological conditions (aOR 1.53, 95% CI 1.48–1.58, *p <* 0.001), gabapentinoid use (aOR 2.52, 95% CI 2.43–2.61, *p <* 0.001), and MME/day at initiation (aOR 1.08, 95% CI 1.07–1.08, *p <* 0.001). After adjustment for case mix, 3 of the 10 UK regions (North West [16%], Yorkshire and the Humber [15%], and South West [15%]), 103 practices (25.6%), and 540 prescribers (3.5%) had a higher proportion of patients with long-term use compared to the population average. This study was limited to patients prescribed opioids in primary care and does not include opioids available over the counter or prescribed in hospitals or drug treatment centres.

**Conclusions:**

Of patients commencing opioids on very high MME/day (≥200), a high proportion stayed in the same category for a subsequent 2 years. Age, deprivation, prescribing factors, comorbidities such as fibromyalgia, rheumatological conditions, recent major surgery, and history of substance abuse, alcohol abuse, and self-harm/suicide were associated with long-term opioid use. Despite adjustment for case mix, variation across regions and especially practices and prescribers in high-risk prescribing was observed. Our findings support greater calls for action for reduction in practice and prescriber variation by promoting safe practice in opioid prescribing.

## Abstract 2

In this analysis of electronic health records, Meghna Jani and colleagues investigate how opioids are prescribed in UK primary care patients.

## Author summary

**Why was this study done?:**

- Whilst opioid prescribing for non-cancer pain has risen in the US and Canada, trends over time are less clear in the UK.
- No studies to our knowledge have evaluated how opioid dose/potency changes over time in UK patients started an opioid for the first time for non-cancer pain, to assess escalation, tapering, and long-term use.
- Physician prescribing behaviour has been implicated as a key driver of rising opioid prescriptions and long-term opioid use; however, this needs to be interpreted in the context of regional and individual patient differences.
- The association of region, practice, prescriber, and individual factors with long-term opioid use is highly important, as this has implications for policy and future targeted public health interventions.

**What did the researchers do and find?:**

- We conducted a study of 1,968,742 new opioid users without cancer from primary care electronic health records across the UK.
- We found that between 2006 and 2017 codeine was the most commonly prescribed opioid: There was a 5-fold increase in codeine prescriptions, a 7-fold increase in tramadol prescriptions, and a 30-fold increase in oxycodone prescriptions for non-cancer pain.
- Prescribing factors (e.g., high dose/potency of opioid or concurrent gabapentinoid use), older age, higher socioeconomic deprivation score, and other conditions including fibromyalgia, rheumatological conditions, history of substance abuse, suicide/self-harm, alcohol abuse, and major surgery were associated with long-term opioid use.
- After accounting for individual patient factors, the North West, Yorkshire and the Humber, and South West regions of England were associated with a higher risk of long-term opioid use.
- Whilst there were only 3.5% of prescribers who had significantly higher prescribing practices leading to long-term use after adjustment of patient factors, where they did, rates were up to 3.5 times higher than the population average.

**What do these findings mean?:**

- Clinicians should take care in prescribing high dose opioids at initiation and closely monitor those with the above individual factors for long-term use.
- The variation observed across regions, practices, and prescribers after adjustment for patient factors supports calls for action to reduce such variation and harmonise prescribing practices.

## Introduction

The sharp increase in prescription opioid use for non-malignant pain in the US, Canada, and several European countries [1–3] has led to concerns of a similar epidemic in the UK. Opioids have now become the leading cause of accidental death and unintentional injury in the US [4]. In the UK, opioid-related deaths have been increasing over the last few decades, the majority of which are non-intentional [3,5,6]. Alongside this, a rise in opioid prescribing, based on national population-level prescribing datasets, has been reported (for all indications including cancer) [7,8]. A recent Public Health England analysis revealed 13% of the UK adult population had 1 or more prescriptions of opioids dispensed between 2017 and 2018 [9].

Opioids are associated with several serious adverse outcomes that are believed to be dose and potency dependent [10]. The escalation rate to higher doses and more potent opioids is likely to also contribute to long-term prescriptions, which in turn may be associated with opioid dependence, addiction, and overdose [11]. Until recently, several commonly prescribed opioids did not have a recommended maximum dose, despite minimal evidence of benefit in non-chronic pain at higher doses. This may lead to considerable variation in opioid prescribing in the context of chronic pain following initiation, including transitioning to stronger opioids, higher dose, or combination opioids, or not reducing dose in a timely manner. The longitudinal opioid pathway of patients commencing opioids for non-cancer pain, the scale of dose escalation/tapering, and ensuing long-term use remain unexplored.

Variation in opioid prescribing across UK regions has been described recently on a population level based on data from clinical commissioning groups [8]. Furthermore, physician prescribing behaviour has been described to be one of the key drivers of rising opioid use [12]. However, the influence of region, practice, and prescriber requires interpretation within their context, by accounting for individual patient characteristics. No studies to our knowledge have investigated the extent to which regions, practices, and individual general practitioners (GPs) vary in opioid prescribing, accounting for the patient (case) mix nor the implications of such variations for long-term opioid prescribing. Identification of what individual patient characteristics are associated with long-term opioid prescribing in primary care would allow prescribers to exercise vigilance and explore alternatives to opioids where appropriate in certain patient subgroups.

The study objectives were to (i) describe trends of the most commonly prescribed opioids for non-cancer pain in UK primary care over a 12-year period (2006–2017) in new users, (ii) assess the transition of morphine milligram equivalents (MME; accounting for dose, opioid type, and sequence of use) in the first 2 years after first prescription, (iii) quantify and identify risk factors for the transition from new user to long-term opioid user, and (iv) quantify the variation of long-term use attributed to region, practice, and prescriber, accounting for patient mix and chance variation.

## Methods

### Data source

We conducted a retrospective observational study from 1 January 2006 to 31 December 2017 using the Clinical Practice Research Datalink (CPRD), a database of anonymised UK primary care electronic health records. In the UK, most patients are registered with a GP, who are often the first point of medical contact and act as ‘gatekeepers’ in the national healthcare system. The majority of opioids in the UK therefore are prescribed in primary care. CPRD collects de-identified patient data from a network of general practices across the UK, providing a longitudinal, representative UK population health dataset. One in 5 practices in the UK contribute data to CPRD through an opt-in system (established for over 30 years). CPRD is one of the largest research databases of longitudinal primary care records in the world and contains information from >14 million registered patients. Prescriptions are recorded electronically, and clinical data including diagnoses are documented using Read codes. Only data that had undergone quality checks by CPRD and were ‘up to standard’ were used in this study. This study is reported as per the Strengthening the Reporting of Observational Studies in Epidemiology (STROBE) guideline (S1 STROBE Checklist).

### Study population

Patients aged ≥18 years without prior cancer who were new users of opioids were identified, in order to establish an incident user cohort prescribed an opioid for non-cancer indications. A 24-month ‘wash-out’ period prior to the index date was used to identify new users. Patients with a previous history of a malignancy Read code up to 10 years prior to the index date were excluded, with the exception of non-melanoma skin cancer. Follow-up start was defined as the date of the first opioid prescription for a given individual (index date). Patients stayed in the cohort until end of follow-up, death, or they left the practice. Patients on methadone were excluded because, in the UK, it is primarily prescribed as an opioid addiction treatment and not consistently prescribed by GPs. S1 Fig describes the derivation of the cohort.

### Covariates

Baseline characteristics such as age, sex, ethnicity, comorbidities that comprise the Charlson Comorbidity Index [13], smoking, and deprivation score were measured using data from the year prior to index date. Socioeconomic status was assessed using linked data for Townsend deprivation score, a composite measure of material deprivation based on UK census data [14]. Townsend deprivation score incorporates 4 variables: unemployment (as a percentage of those aged ≥16 years who are economically active), non-car ownership (as a percentage of all households), non-home ownership (as a percentage of all households), and household overcrowding.

To identify risk factors for long-term opioid use, we identified additional a priori variables, based on clinical knowledge and published literature. All diagnoses were identified using Read codes 1 year prior to first opioid prescription. The US Centers for Disease Control and Prevention (CDC) has identified certain factors associated with opioid misuse such as previous substance use disorder, major depression, and use of psychotropic medications [15], which we defined in CPRD. Psychotropic medications included antiepileptics, antihistamines, antiparkinsons, antipsychotics, anxiolytics, hypnotics, and sedatives. Other factors evaluated included prior history of suicide or self-harm, alcohol excess, major surgery in the last 1 year, and pain conditions such as back pain, migraine, and fibromyalgia. Rheumatological disorders included rheumatoid arthritis, systemic lupus erythematosus, myositis, and giant cell arteritis (defined by Charlson Comorbidity Index score [13]). The use of psychotropic medications, benzodiazepines, and gabapentinoids was defined as any use in the 1 year prior to the index date, including the date the first opioid was prescribed. Prescriber, general practice, and regional information for each patient was obtained to examine variation at each level in opioid prescribing.

### Opioid drug preparation and exposure

Opioid exposure data were prepared using a drug preparation algorithm published previously [16]. The decisions made to prepare the data are described in S2 Fig. Classes of opioids were divided into weak opioids (codeine, dihydrocodeine, meptazinol), moderate opioids (tramadol, tapentadol), and strong opioids (morphine, oxycodone, fentanyl, buprenorphine, diamorphine, hydromorphone, pethidine). Tramadol and tapentadol were classed as moderate-strength opioids, as despite their low MME they are phenotypically distinct from conventional weak opioids due to their dual mechanism as a partial serotonin-norepinephrine reuptake inhibitor. Combination formulations, such as co-codamol, were classed according to their active opioid ingredient. If patients were on ≥1 opioid at index, we categorised them into a separate combination opioids group.

To allow direct comparison of doses and opioid potencies across different drugs and formulations we calculated MME for each prescription. MME/day was defined as the daily dose for each prescription multiplied by the equivalent analgesic ratio as specified by the CDC [15]. For transdermal buprenorphine and fentanyl formulations, strength per hour and the duration of delivery rate of the formulation was considered in the dose calculation to avoid underestimation of daily MME. An episode of long-term opioid use was defined as at least 3 opioid prescriptions issued within a 90-day period, or ≥1 opioid prescription lasting at least 90 days, in the first year of follow-up, not including the first 30 days after the index date. When defining long-term use, we ignored the first 30 days following the index date to allow for acute pain treatment.

### Statistical analysis

Descriptive statistics were used to assess the baseline characteristics of the cohort, stratified according to opioid strength at initiation. Although a prospective analysis plan has not been included, the objectives of this study were determined at the outset of the planned study according to unmet need in the literature/clinical relevance and were not adapted subsequently. No data-driven changes to the analyses took place after obtaining the data during the statistical analysis stage.

#### Prescribing trends over time

To evaluate trends of opioid prescribing over time, the rate of prescription for each opioid drug was calculated by calendar year by dividing the number of prescriptions per year for the cohort (numerator) by the number of eligible patients registered in CPRD per year (denominator). Raw denominator numbers of patients registered were provided by CPRD in April 2018 and prepared for use (S1 Fig).

#### Transition of opioids over 2 years

Patients were stratified into 4 categories according to the average MME/day in the first 6 months after index date to incorporate the type, potency, and dose of the opioid. MME categories were as follows: low, <50 MME/day; medium, 50–119 MME/day; high, 120–199 MME/day; and very high, ≥200 MME/day. For instance, a prescription of 30 mg codeine 4 times a day would equal 18 MME/day. An oxycodone prescription of 40 mg 4 times/day equates to 240 MME/day. In patients on a combination of opioids, MME was calculated for each drug, and the sum was taken as the MME/day. Stacked plots and Sankey diagrams were created to quantify visually the sequential transition of MME/day, in 6-month bands, over a 2-year time window from the index date. One plot was generated for each of the MME dosage categories derived from the first 6 months’ exposure (low, medium, high, and very high).

#### Transition from new user to long-term opioid user

A multi-level random-effects logistic regression model was used to examine the association of different patient characteristics with the odds of becoming a long-term opioid user. Person-level characteristics investigated include age, sex, ethnicity, deprivation score, and the comorbidities outlined above. More details about the approach are provided in S1 Appendix. To examine opioid variation amongst prescribers, general practices, and regions after adjusting for patient case mix, we used a nested random-effects structure (i.e., prescribers nested within practices and practices nested within regions). The approach introduced by Snijders and Bosker [17] was followed to obtain the explained variation at each level of the hierarchy. Furthermore, the posterior distributions of the prescriber-, practice-, and region-level random effects were simulated using the REsim function in the merTools package [18] for the fully adjusted models. The adjusted random-effects estimates along with 95% confidence intervals were then ranked and plotted on an odds ratio (OR) scale as well as percentage value. To express the adjusted estimates as a proportion, we used the transformation described in S1 Appendix. ORs with the lower end of the 95% CI > 1 were associated with a higher risk, and ORs with the upper end of the 95% CI < 1 were associated with a lower risk, of long-term opioid use. ‘High-risk’ regions, practices, or prescribers were defined as those where the entire adjusted 95% CI lay above the population average (i.e. 1). The risk of becoming a long-term opioid user attributed to a specific practice was then plotted against the proportion of high-risk prescribers within each practice to evaluate the influence of a high-risk prescriber on the practice (S5 Fig). All analyses were performed in STATA version 14.0 and R version 3.5.0.

#### Ethics

The study was approved by the CPRD’s Independent Scientific Advisory Committee (approval number: 16_278).

## Results

We identified 1,968,742 new users of opioids who met our inclusion criteria, of which 88.2% were initially commenced on a weak opioid, 8.5% on a moderate opioid, 2.6% on a strong opioid, and 0.7% on combination opioids (Table 1). The highest proportion of new opioid users for the weak, moderate, and combination opioid categories were aged 35–54 years, whereas patients who were started on strong opioids were older: 31.5% of strong opioids were prescribed to patients ≥85 years (compared to 4.1% and 3.3% in the weak and moderate opioid groups, respectively). Proportionally more patients commencing strong opioids had Charlson Comorbidity Index score ≥ 4 (7.5% compared to <2% in other opioid groups). Townsend deprivation quintiles were represented in similar proportions across weak, medium, strong, and combination opioids. The proportion of patients on all types of opioids was slightly lower in the most deprived category, between 11% and 16%. The strong opioid group had the highest proportions of patients on prior benzodiazepine, gabapentinoid, or psychotropic medications (Table 1).

**Table 1:** Baseline characteristics of new users of opioids (*n* = 1,968,742) grouped by opioid strength at initiation.

Characteristic	Monotherapy at index			Combination therapy at index
	Weak opioids (*n* = 1,736,398; 88.2%)	Moderate opioids (*n* = 166,524; 8.5%)	Strong opioids (*n* = 51,126; 2.6%)	Combination opioids (*n* = 14,694; 0.7%)
**Age, years, mean (SD)**	50.5 (19.0)	51.1 (17.6)	69.6 (21.0)	54.9 (19.9%)
**Age group (years), *n* (%)**				
18–24	155,350 (9.0)	10,175 (6.1)	1,061 (2.1)	739 (5.0)
25–34	262,288 (15.1)	22,238 (13.4)	3,060 (6.0)	1,778 (12.1)
35–44	300,793 (17.3)	31,705 (19.0)	4,089 (8.0)	2,525 (17.2)
45–54	300,381 (17.3)	33,763 (20.3)	4,942 (9.7)	2,715 (18.5)
55–64	273,806 (15.8)	28,946 (17.4)	5,418 (10.6)	2,314 (15.8)
65–74	218,957 (12.6)	20,662 (12.4)	6,228 (12.2)	1,773 (12.1)
75–84	153,738 (8.9)	13,515 (8.1)	10,206 (20.0)	1,341 (9.1)
≥85	71,085 (4.1)	5,520 (3.3)	16,122 (31.5)	1,509 (10.3)
**Female, *n* (%)**	991,322 (57.1)	92,675 (55.7)	31,913 (62.4)	8,178 (55.7)
Ethnicity, where reported*, ***n* (%)**				
White	1,114,790 (89.0)	115,023 (91.4)	37,116 (94.6)	10,016 (92.1)
Asian	51,499 (4.1)	3,479 (2.8)	596 (1.5)	259 (2.4)
Black	29,231 (2.3)	1,983 (1.6)	307 (0.8)	144 (1.3)
Other	18,979 (1.5)	1,437 (1.1)	266 (0.7)	111 (1.0)
Mixed	10,794 (0.9)	892 (0.7)	164 (0.4)	71 (0.7)
Unknown	27,812 (2.2)	2,998 (2.4)	765 (2.0)	276 (2.5)
Townsend deprivation score, where reported*, ***n* (%)**				
Q1 (least deprived)	193,601 (20.8)	19,180 (21.0)	6,539 (21.9)	1,632 (21.6)
Q2	194,478 (20.9)	20,320 (22.2)	7,651 (25.6)	1,705 (22.5)
Q3	194,884 (21.0)	19,004 (20.8)	6,729 (22.5)	1,630 (21.5)
Q4	198,714 (21.4)	19,285 (21.1)	5,766 (19.3)	1,565 (20.7)
Q5	147,054 (15.8)	13,599 (14.9)	3,182 (10.7)	1,041 (13.7)
**Charlson Comorbidity Index, *n* (%)**				
Low score (0)	1,249,477 (72.0)	117,614 (70.6)	25,101 (49.1)	9,732 (66.2)
Medium score (1–3)	454,906 (26.2)	45,652 (27.4)	22,181 (43.4)	4,527 (30.8)
High score (≥4)	32,015 (1.8)	3,258 (2.0)	3,844 (7.5)	435 (3.0)
**Conditions associated with opioid dependency, *n* (%)**				
History of alcohol dependency	37,787 (2.2)	4,496 (2.7)	1,162 (2.3)	452 (3.1)
History of substance use disorder	25,851 (1.5)	3,558 (2.1)	1,702 (3.3)	374 (2.6)
History of depression	374,209 (21.6)	40,350 (24.2)	10,756 (21.0)	3,440 (23.4)
History of suicide and self-harm	57,119 (3.3)	7,265 (4.4)	1,834 (3.6)	815 (5.6)
**Concomitant medication, *n* (%)**				
Baseline benzodiazepine use^†^	156,050 (9.0)	20,252 (12.2)	14,319 (28.0)	2,806 (19.1)
Baseline gabapentinoid use^†^	22,627 (1.3)	6,495 (3.9)	4,054 (7.9)	1,235 (8.4)
Baseline psychotropic drug use^†^	197,133 (11.4)	24,996 (15.0)	18,752 (36.7)	3,654 (24.9)
**Major surgery, *n* (%)**	41,088 (2.4)	10,490 (6.3)	2,354 (4.6)	1,231 (8.4)
Smoking status, where reported*, ***n* (%)**				
Never	341,075 (45.5)	30,060 (42.3)	9,708 (49.2)	2,601 (44.0)
Former	220,141 (29.4)	21,392 (30.1)	6,614 (33.5)	1,672 (28.3)
Current	188,185 (25.1)	19,671 (27.7)	3,423 (17.3)	1,639 (27.7)
**Missing, *n* (%)**				
Ethnicity	483,293 (27.8)	40,712 (24.5)	11,912 (23.3)	3,817 (26.0)
Townsend deprivation score	807,667 (46.5)	75,136 (45.1)	21,259 (41.6)	7,121 (48.5)
Smoking status	986,997 (56.8)	95,401 (57.3)	31,381 (61.4)	8,782 (59.8)

Proportions are presented as percentage of non-missing data.

*Some patients had missing data for certain variables, which is reported at the end of the table.

^†^Baseline use refers to use 1 year prior to and including the index date.

Q, quintile; SD, standard deviation.

### Population-level opioid prescribing patterns

**Fig 1:**
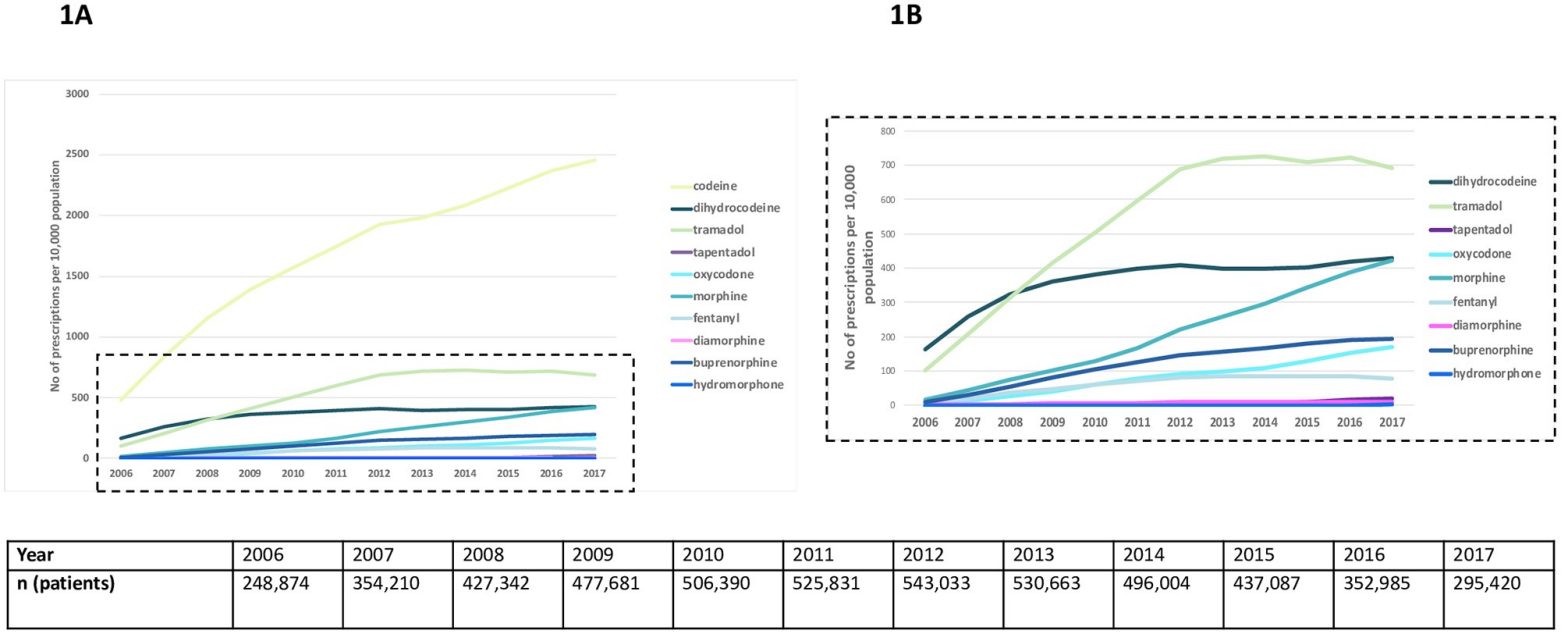
Opioid utilisation in the Clinical Practice Research Datalink by individual opioid. (A) The most frequently prescribed opioids, 2006–2017. (B) All opioids in (A) except for codeine.

The most commonly used opioids were codeine, dihydrocodeine, and tramadol. Over a 12-year period, 2006–2017, codeine use increased 5-fold, from 484 to 2,456 prescriptions per 10,000 population/year. Dihydrocodeine, tramadol, and fentanyl prescriptions increased between 2006 and 2012, and plateaued thereafter until end of 2017. Within the strong opioids group, oxycodone prescribing rose approximately 30-fold, from 5 to 169 prescriptions per 10,000 population/year over 12 years. Morphine prescriptions also rose considerably, from 18 to 422 prescriptions per 10,000 population/year between 2006 and 2017 (Fig 1).

**Fig 2:**
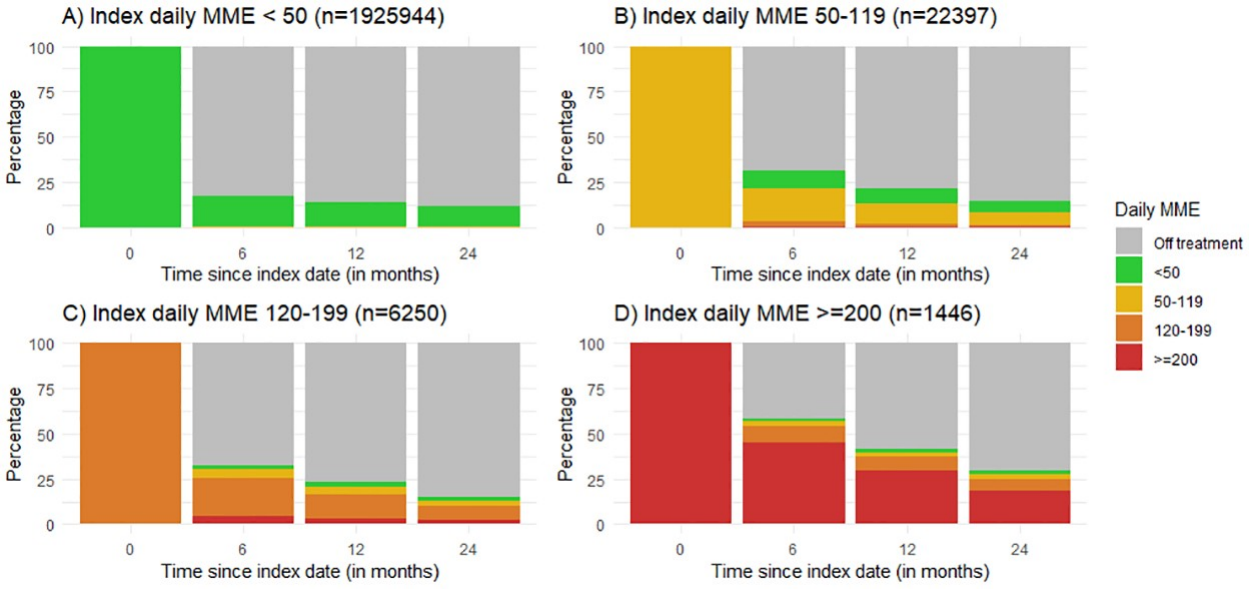
Transition of MME dosage categories over a 2-year period from index date, stratified by dose category in the first 6 months. Each panel represents the index daily morphine milligram equivalents (MME). Each bar represents the proportion of patients that transition to a different level of MME, stay in the index MME category, or come off treatment over the 2 years of follow-up. The MME value thresholds were chosen considering differences in recommendations between international guidelines. In the UK, the Faculty of Pain Medicine suggests harms outweigh benefits when patients exceed 120 MME/day [27].

The transition of MME dose in new users of opioids over a 2-year period is shown in Fig 2. The majority of patients were started on <50 MME/day (*n =* 1,925,944), of whom 1,706,574 (88.6%) had stopped completely at 2 years and 219,073 (11.4%) had continued in the same category or higher at 2 years. Of the 24,315 patients who started on a medium MME (50–119 MME/day) as their starting dose, by 1 year 3,249 (13.4%) had escalated to a higher MME group, whilst 21,066 (86.7%) were tapered to <50 MME/day (including discontinuation). At 2 years 1,584 (6.5%) had stayed in the same MME category, and 358 (1.5%) had escalated to a higher MME category. Of the patients commencing on very high MME (≥200 MME/day; *n =* 1,446 [0.08%]), 656 (45.4%) continued to be on very high MME/day at 6 months, 434 (30.0%) at 1 year, and 270 (18.7%) at 2 years. Transitions in the form of Sankey diagrams and actual patient numbers at 6, 12, and 24 months are presented in S3 Fig.

### Variation of long-term opioid use by prescriber, practice, and region

**Fig 3:**
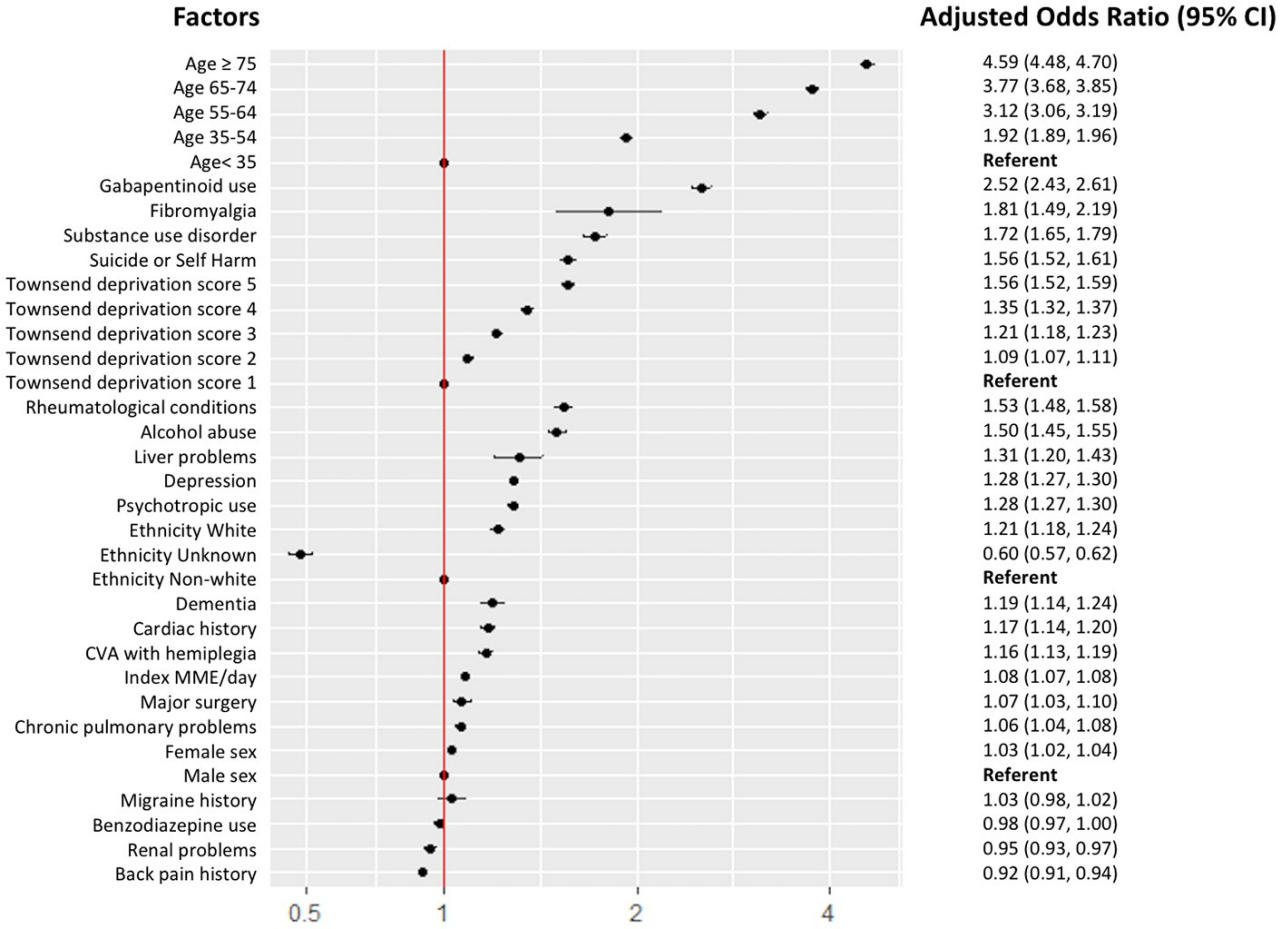
Factors associated with long-term opioid use using a multi-level model accounting for clustering of individuals within prescriber, practice, and region. CVA, cerebrovascular accident; MMI, morphine milligram equivalents.

In our new user cohort, 14.6% became long-term opioid users in the first year after the index date. In the fully adjusted model, a number of individual factors were identified as being associated with a higher odds of long-term opioid use including older age, social deprivation, fibromyalgia, suicide/self-harm, excess alcohol, gabapentinoid use, psychotropic use, major surgery, and initial dose (Fig 3) (all *p-*values < 0.001). The strongest association was seen in those who were ≥75 years, who were 4.6 (95% CI 4.5 to 4.7, *p <* 0.001) times more likely to become a long-term opioid user compared to those who were <35 years.

**Fig 4:**
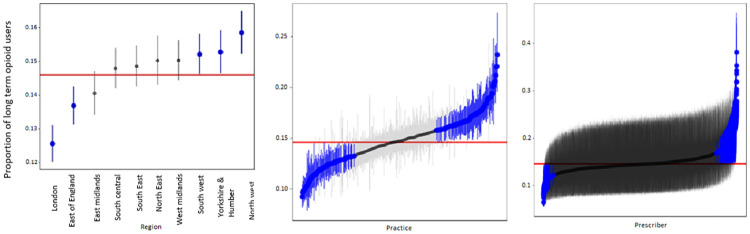
Level of variation among regions, practices, and prescribers in terms of the proportions of new opioid users with long-term opioid use. Each horizontal line represents the point estimate with 95% confidence interval by region (left) or practice (middle) or prescriber (right). Regions, practices, and prescribers with 95% confidence intervals entirely above or below the population average (red vertical line) are indicated in blue. For instance, the adjusted proportion of long-term users for the North West region (15.8%) is significantly higher than the population average (14.6%). The largest variation is seen among practices and prescribers. The proportion of long-term opioid users for some practices reached up to 23.2%. The proportion of long-term opioid users for the highest risk prescriber was 37.2%.

Fig 4 illustrates the prescriber-, practice-, and regional-level variation in the odds of long-term opioid use. After adjustment for case mix, there was still considerable variation among regions, practices, and prescribers. Prescribers explained 2.2% of the total variation whilst practices and regions explained 0.6% and 0.01% of the variation, respectively. Three regions (namely North West, Yorkshire and the Humber, and South West) had proportions of long-term opioid users significantly greater than the population average (Fig 4). The proportion of long-term users for North West, Yorkshire and the Humber, and South West was 16% (95% CI 15%–17%), 15% (95% CI 15%–16%), and 15% (95% CI 15%–16%), respectively, while the proportion of long-term users in London was 13% (95% CI 12%–13%). High levels of variation were observed among practices after case mix adjustment. Of the 402 practices included in the study, 103 practices (25.6%) were associated with a significantly higher risk of long-term opioid use (Fig 4). The proportion of long-term users for the most high-risk practice was 23% (95% CI 19%–28%) (OR 1.79, 95% CI 1.42–2.26), while the proportion of long-term users for the least at-risk practice was 10% (95% CI 9%–11%) (OR 0.60, 95% CI 0.53–0.68) (S4 Fig). After case mix adjustment, 540 (3.5%) prescribers were associated with a significantly higher risk of long-term opioid use. This small proportion of high-risk prescribers had notably high prescribing behaviour, with the odds of a new opioid user becoming a long-term user reaching up to an OR of 3.56 (95% CI 2.53–5.02) compared to the population average (Fig 4). This equated to around 37% of new users for the highest risk prescriber becoming long-term users by the end of the first year. In certain high-risk practices, the propensity of the practice being high-risk was driven by a few prescribers (S5 Fig).

## Discussion

In this large national cohort of opioid-naïve patients in CPRD, we found a substantial increase in opioid prescribing for non-cancer pain between 2006 and 2017. Of the patients who started on high (120–199 MME/day) or very high dose opioids (≥200 MME/day), 10.3% and 18.7%, respectively, remained in the same MME/day category or higher at 2 years. We identified a number of patient-specific factors associated with long-term opioid use not previously identified in a UK population, most notably high initial dose/potency of opioid, fibromyalgia, rheumatological conditions, history of depression, prior gabapentinoid/psychotropic use, and history of major surgery. A wide variation in the risk of long-term opioid use was observed by prescriber, practice, and region. In addition, a regional divide in long-term opioid use risk was found, with the North West, Yorkshire and the Humber, and South West regions associated with the highest levels of long-term opioid use. Whilst there was a small proportion of prescribers (3.5%) who had significantly higher prescribing practices, their opioid prescribing rates were considerably higher in comparison to the population average. After adjusting for case mix, certain prescribers within a practice could be observed to be driving their entire practice towards high long-term opioid prescribing.

### Comparison with previous studies and interpretation

To our knowledge this is the largest UK study evaluating opioid prescribing for non-cancer pain, with patient-level data to ascertain total amount of drug prescribed in terms of MME/day. We addressed a number of key questions quantifying the variance in prescribing at the regional, practice, and prescriber level. The finding of an overall rise in opioid prescribing is complementary to a recent study using National Health Service (NHS) digital pharmacy claims data demonstrating a 34% increase in opioid prescriptions between 1998 and 2016 [7]. This study however included prescription data on all opioids, including those prescribed for cancer pain, and lacked individual-level data, and high dose MME definitions were based on presumptions of daily dose. Our results are consistent with a previous cross-sectional CPRD study that reported a rise in morphine, oxycodone, fentanyl, and buprenorphine prescribing between 2000 and 2010 in a non-cancer population that we were able to extend both in time frame and in the range of opioids included [19]. In our study, codeine, morphine, and buprenorphine prescriptions in particular continued to rise until the end of 2017. Since 2013 in the UK, national regulations have been designed to improve the use and monitoring of controlled drugs such as opioids [20]. An increase in tramadol, oxycodone, and fentanyl prescriptions continued until 2012, following which prescribing plateaued, suggesting that GPs may have already started to reduce use of new opioids for these medications earlier. However, such regulations did not change prescribing patterns for morphine or buprenorphine.

Clinicians have an opportunity to be vigilant about what type of patient may become a long-term opioid user. A number of individual features associated with increased odds of long-term opioid use were identified. Older age and social deprivation were associated with an incremental increase in risk of long-term opioid use (Fig 3). Clinical-commissioning-group-level deprivation has been associated with higher population-level opioid prescribing using NHS digital data [8]. In the US, substance abuse, depression, and psychotropic medicines have been associated with an increased risk of opioid misuse [15,21], and we found these factors to also be associated with an increased risk of long-term opioid use in opioid-naïve patients in the UK. Additionally, benzodiazepines and gabapentinoid use also significantly increased odds and may be a surrogate for chronic pain severity. Concomitant use with opioids has also been associated with an increased risk of death [22,23]. Additionally, alcohol excess, fibromyalgia, rheumatological conditions, diabetes, and prior major surgery were significantly associated with a higher odds of long-term opioid use. In the US, new opioid users, especially post-surgery, have been shown to be a vulnerable population both for new persistent use and for developing opioid dependence/overdose [24,25]. Therefore, addressable patient-level factors and the existence of certain vulnerable groups at a higher risk of long-term use warrant increased awareness in prescribing clinicians.

An important finding in our study was that 14.6% of new users became long-term users over a 1-year period. In patients who were started on high doses of opioids (≥120 MME/day), a considerable proportion continued on higher doses throughout the following year. We also observed a wide variation at the practice and prescriber level in the adjusted odds of a new opioid user becoming a long-term opioid user (Fig 4), and the propensity of a practice being ‘high risk’ for its patients becoming long-term opioid users was being driven by a few prescribers in some cases. Of the 3 levels, prescribers had a bigger influence on long-term use than practice or region in our study. Whilst variation in prescribing between provider and practice has not been explored previously within a national UK setting, a US study examined the extent to which emergency physicians varied in rates of opioid prescribing and the implications of that variation for long-term opioid use. It was reported that prescribing rates varied widely between low-intensity and high-intensity prescribers (7.3% versus 24.1%), with long-term opioid use significantly higher among patients treated by high-intensity prescribers [26].

There are a few possibilities why prolonged opioid use may occur, in addition to ongoing appropriate prescribing for patients with clinical need. The variability in prescribing may in part be explained by unclear guidance regarding best practice in managing non-cancer pain. The advice regarding MME/day thresholds beyond which tapering should occur varies internationally [15,27]; therefore, GPs may not be aware of which patients to intervene with. Currently there is considerable heterogeneity in guidance internationally regarding dose thresholds that warrant caution, which vary between 50 and 200 MME/day [15,28,29]. In the US, national guidelines advise precautions and reassessment of patients exceeding 50 MME per day, and that prescribers should avoid increasing dose to 90 MME or more per day [15]. The Faculty of Pain Medicine in the UK suggests harms outweigh benefits when patients exceed 120 MME/day [27]. There is minimal guidance based on scientific evidence on how best to reduce/discontinue opioids in chronic pain. Tapering could fail to happen because clinicians are guided by patients, who may understandably fear worsening pain or withdrawal symptoms, may lack adequate social/healthcare support, or could perceive a lack of effectiveness of non-opioid pain relief options [30]. Alternatively, transition to long-term opioid use could be driven by ‘clinical inertia’ in some instances [26], where prescribers continue providing repeat prescriptions, assuming drug effectiveness without regular review.

The adjusted odds for long-term opioid use in opioid-naïve patients was highest in the North West, Yorkshire and the Humber, and South West regions of England (Fig 4). Regional UK variation in population-level opioid prescribing between the North and the South of England has been observed in recent studies [7,8]. A previous study using NHS digital data showed that 9 out of 10 of the highest prescribing areas in the country were located in the North of England, and there was an association with social deprivation [8]. Health is known to be worse in the North of England, and a strength of the present study was that we were able to account for case mix also, while previous studies have not. Whilst chronic pain severity was not measured, there is no known significant regional variation in the prevalence of chronic pain across strategic health authorities [31]. Therefore, it should not account for the observed regional differences in long-term opioid prescribing. We found, even after adjusting for deprivation (which has been linked to chronic pain [31]), the North of England/South of England disparities in long-term opioid use continued to exist.

### Limitations of this study

In the UK, codeine and dihydrocodeine were available over the counter during the study period. Because CPRD data capture electronic prescription data from primary care physicians, the findings likely underrepresent overall drug utilisation of weaker opioids. In 2014 tramadol was reclassified as a schedule 3 drug, and prescriptions longer than 1 month prohibited at any one time. Therefore, the rise in prescriptions may reflect shorter prescriptions for certain medications. The advantage of using this measure is it allows for comparisons with other studies internationally. Treatments for opioid addiction are mainly prescribed through specialist addiction centres in the UK, rather than through primary care, and are thus not available in the dataset. We were therefore not able to account for these in the analysis.

Measuring opioid exposure using prescription records is complicated by the possibility that patients may not fill their prescriptions, medications are administered by the patient, medications can be taken as required, or there may be issues around divergence, none of which are captured in primary care databases. Since data for this study were collected as part of routine clinical care, we did not have access to patient-level pain scores, severity of underlying diseases, or patient perceptions of opioid prescribing. We did however adjust for benzodiazepine, gabapentinoid, and psychotropic drug use, which could be used as a potential proxy for pain severity.

### Conclusions

In conclusion, overall opioid prescribing has increased over 12 years, with wide variation across the UK after adjusting for patient characteristics. There were considerable differences within practices/prescribers in opioid prescribing and the associated risk of long-term opioid use, even after adjusting for case mix. Patients started on high MME were more likely to stay in the same category for the following 2 years. Whilst reasons are likely to be multi-factorial, exercising vigilance when prescribing to those with identified individual risk factors and improved educational interventions to improve clinical decision making are likely to be beneficial. Our findings are important as they offer a potential lever for changing prescribing behaviour and providing interventions in subgroups of patients at higher risk of long-term use. On a practice level, guidance on regular review and dose reduction, as well as using prescriber and practice variations as a proxy for quality of care through audit and feedback, to highlight unwarranted variation to prescribers, could help drive safer prescribing.

## Supporting information

S1 Appendix Detailed description of statistical methods. (DOCX)

S1 Fig Flowchart for CPRD opioid study cohort creation (12-year study window). (DOCX)

S2 Fig Decisions made for drug preparation to derive daily dose. (DOCX)

S3 Fig Sankey diagrams with transitions of MME over 2 years. (DOCX)

S4 Fig Level of variation among regions, practices, and prescribers in terms of the odds of long-term opioid use. (A) Regions. (B) Practices. (C) Prescribers.
(DOCX)

S5 Fig Risk of long-term opioid use for an individual in relation to prescriber and practice. (DOCX)

S1 STROBE Checklist (DOCX)

## Abbreviations

aOR: adjusted odds ratio
CDC: Centers for Disease Control and Prevention
CPRD: Clinical Practice Research Datalink
GP: general practitioner
MMI: morphine milligram equivalents
NHS: National Health Service
OR: odds ratio
