# Supplementary material for: Time trends and prescribing patterns of opioid drugs in UK primary care patients with non-cancer pain: A retrospective cohort study
Source: PLoS Med. 2020 Oct 15;17(10):e1003270. doi: 10.1371/journal.pmed.1003270 (PMC7561110; doi:10.1371/journal.pmed.1003270)
Supplement: S1 Appendix — (DOCX) [file pmed.1003270.s001.docx]

# S1 Appendix: Detailed description of statistical methods

**Multi-level logistic regression model for long –term opioid use**

**Model definition**

Given our interest in contextual effects, we considered a hierarchical data structure where patients (level-1) were nested within prescribers (level-2), prescribers nested within practices (level-3) and practices nested within regions (level-4). Our aim was to determine whether there were a significant prescriber, practice and regional variation in the odds of long-term opioid use and, if so, whether those clusters (hierarchy) accounted for such variation net of patients’ characteristics (case mix). We adopt a multi-level modelling approach assuming a nested random effect structure corresponding to prescriber, practice and region to estimate the proportion of variance in individual outcomes that is attributed to each of the three levels: prescriber, practice, and region. Let $y_{ijkl}$ denote the long-term opioid use status (1= if yes and 0 if not) for the $i^{th}$subject registered to the $j^{th}$prescriber belonging to the $k^{th}$practice in the $l^{th}$region. The multi-level logistic regression model (1) is then defined as,

$$y_{ijkl}\sim Binomial\left( 1, \pi_{ijkl} \right),$$

$$logit\left( \pi_{ijkl} \right)=\beta_{0}+\boldsymbol{\beta}^{\boldsymbol{'}}\boldsymbol{X}+b_{l}+v_{kl}+u_{jkl},$$

where $b_{l}\sim N(0, \sigma_{b}^{2})$ is a region specific random effect, $v_{kl}\sim N(0,\sigma_{v}^{2})$ is practice specific random effect nested within region, and $u_{jkl}\sim N(0, \sigma_{u}^{2})$ is prescriber specific random effect nested within practice and region, $\beta_{0}$ is the intercept and $\boldsymbol{\beta}$ is the vector of coefficient’s corresponding to the vector of risk factors $\boldsymbol{X}$ (including age, sex, comorbidities etc.).

**Explained variance and ICC**

After fitting the multi-level logistic model, we compute several measures to quantify the proportion of variance explained (2). First, we compute the marginal $R_{marginal}^{2}$ that accounts for the variance explained by the fixed factors (risk factors) and the conditional $R_{conditional}^{2}$ for the variance explained by both fixed and random factors as follow:

$$R_{marginal}^{2}=\frac{\sigma_{f}^{2}}{\sigma_{f}^{2}+\sigma_{b}^{2}+\sigma_{v}^{2}+\sigma_{u}^{2}+\frac{\pi^{2}}{3}},$$

$$R_{conditional}^{2}=\frac{\sigma_{f}^{2}+\sigma_{b}^{2}+\sigma_{v}^{2}+\sigma_{u}^{2}}{\sigma_{f}^{2}+\sigma_{b}^{2}+\sigma_{v}^{2}+\sigma_{u}^{2}+\frac{\pi^{2}}{3}},$$

where $\sigma_{f}^{2}$ is the variance of the fixed effect part only. Then, the proportion of variance at the group level is given by the intraclass correlation coefficient (ICC) corresponding to the level of clustering (in this case, prescriber, practice and region).

$$ICC_{prescriber}=\frac{\sigma_{u}^{2}}{\sigma_{b}^{2}+\sigma_{v}^{2}+\sigma_{u}^{2}+\frac{\pi^{2}}{3}}$$

$$ICC_{practice}=\frac{\sigma_{v}^{2}}{\sigma_{b}^{2}+\sigma_{v}^{2}+\sigma_{u}^{2}+\frac{\pi^{2}}{3}}$$

$$ICC_{region}=\frac{\sigma_{b}^{2}}{\sigma_{b}^{2}+\sigma_{v}^{2}+\sigma_{u}^{2}+\frac{\pi^{2}}{3}}$$

**Identification of ‘High risk’ regions, practice and prescribers**

In order to identify prescribers, practices and regions with a higher proportion of long-term opioid users, the posterior distributions of the prescriber, practice and region level random effects were simulated using the **REsim** function in the **merTools** package (Ref-25 in the original article) from the fully adjusted models. The adjusted random effect estimates along with the 95% confidence interval were then ranked and plotted on an odds ratio (OR) scale. To express the adjusted estimates as a proportion, we use the following transformation. For example, for the $l$th region, the estimated proportion of long term users is given by

$$\hat{p_{l}}=\frac{\exp\left( \hat{\beta}_{0}+\hat{b}_{l} \right)}{1+exp(\hat{\beta}_{0}+\hat{b}_{l})},$$

while in odds ratio scale is $exp(\hat{b}_{l})$.

**References:**

1. Breslow N.E, Clayton D.G. Approximate Inference in Generalized Linear Mixed Models. Journal of the American Statistical Association. 1993; 88:421, 9-25
2. Nakagawa S, Johnson P, Schielzeth H. The coefficient of determination R2 and intra-class correlation coefficient from generalized linear mixed-effects models revisted and expanded. J. R. Soc. Interface. 2017 Sep;14(134). pii: 20170213.
