# Supplementary material for: Time trends and prescribing patterns of opioid drugs in UK primary care patients with non-cancer pain: A retrospective cohort study
Source: PLoS Med. 2020 Oct 15;17(10):e1003270. doi: 10.1371/journal.pmed.1003270 (PMC7561110; doi:10.1371/journal.pmed.1003270)
Supplement: S1 Fig — (DOCX) [file pmed.1003270.s002.docx]

**S1 Fig: Flowchart for CPRD Opioid study cohort creation (12-year study window)**

All patients with an Opioid Rx **ever** in CPRD (since records began in 1987 until download on 14.09.2018)

**n = 3,711,928**

Opioid ever-users without a Rx in the 12-year study window (01.01.2006-31.12.2017)

n = 1,102,042

All patients with an opioid Rx in the 12-year study window (01.01.2006-31.12.2017)

**n = 2,609,886**

All prevalent users + under-18 year old new users at index

n = 390,536

Adult new users of opioids in 12-year study window – 01.01.2006-31.12.2017

**n = 2,219,350 (n = 1,200,456 with hes linkage)**

Adult new users failing to satisfy cohort entry date rules

n = 101,464

Adult new users of opioids meeting CPRD cohort entry date rules i.e: crd, uts, lcd, death date, tod

**n = 2,117,886**

Adult new users with cancer Read code in 10 years prior to index

n = 143,325

Adult new users without cancer Read code **in 10 years** prior to index

**n = 1,974,561**

Adult methadone users; n = 5,765

Adult new users (excluding methadone users) without cancer Read code **in 10 years** prior to index

**n = 1,968,796**

Adult new users dropped from cohort after applying Drug Preparation Algorithm

n = 54

Adult new users without cancer Read code **in 10 years** prior to index & post drug preparation

**n = 1,968,742**

*Definition of a new user = opioid free for two years prior to incident prescription. Abbreviations: crd, date the patient’s current period of registration with the practice began; lcd, date of the last collection of data for the practice; hes, hospital episode statistics; tod; date the patient transferred out of the practice; uts, up to standard/ date at which the practice data was deemed to be of research quality

**Derivation of CPRD denominator figures:**

For each of the 12 years, the denominator was derived by starting with all acceptable patients in CPRD, and thereafter excluding those from practices:

(i) that were up to standard after x-year or (ii) with a last collection date before x-year or (iii) had a current registration date after x-year or (iv) who died/transferred out before x-year.
