# Supplementary material for: Time trends and prescribing patterns of opioid drugs in UK primary care patients with non-cancer pain: A retrospective cohort study
Source: PLoS Med. 2020 Oct 15;17(10):e1003270. doi: 10.1371/journal.pmed.1003270 (PMC7561110; doi:10.1371/journal.pmed.1003270)
Supplement: S2 Fig — (DOCX) [file pmed.1003270.s003.docx]

**S2 Fig: Decisions made for drug preparation to derive daily dose**


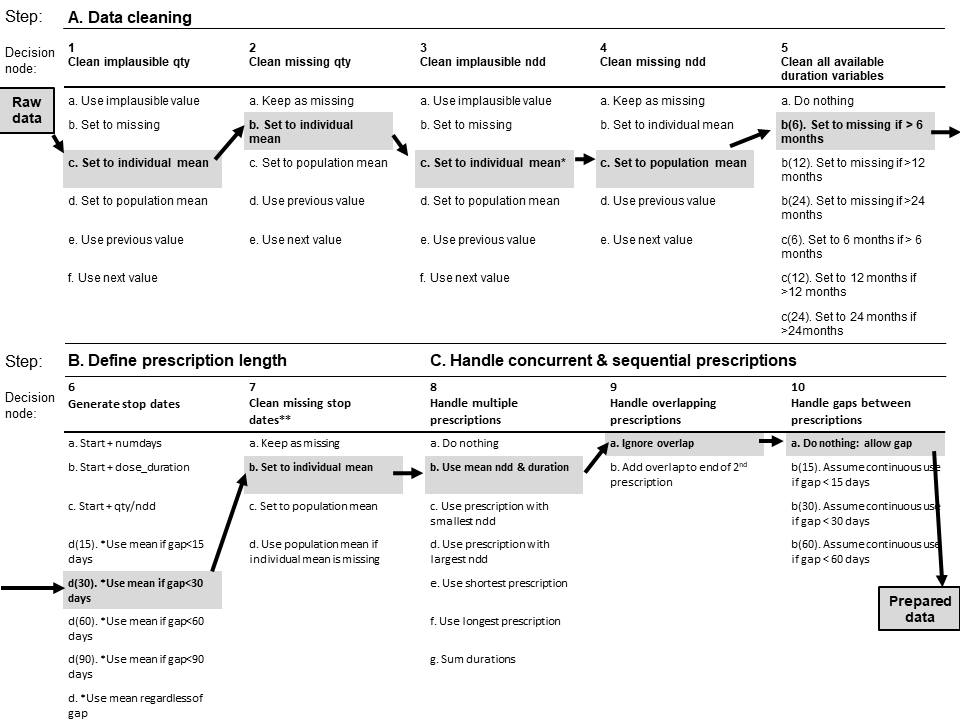


Adapted from reference [23]. *Decision 3c: We set to mean for individual's prescriptions for that drug; if missing set to mean for practice's prescriptions for that drug; if not available set to mean for populations' prescriptions for that drug.
