# Supplementary material for: Time trends and prescribing patterns of opioid drugs in UK primary care patients with non-cancer pain: A retrospective cohort study
Source: PLoS Med. 2020 Oct 15;17(10):e1003270. doi: 10.1371/journal.pmed.1003270 (PMC7561110; doi:10.1371/journal.pmed.1003270)
Supplement: S3 Fig — (DOCX) [file pmed.1003270.s004.docx]

**S3 Fig: Sankey diagrams with transitions of MME over 2-years**


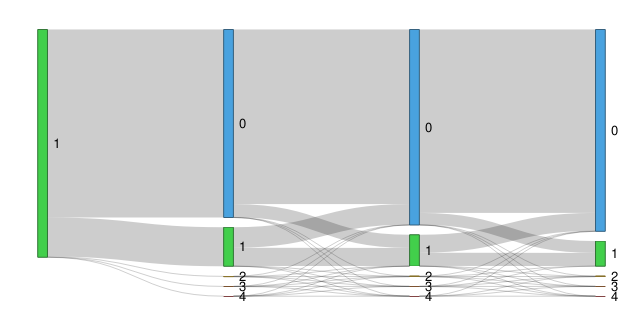

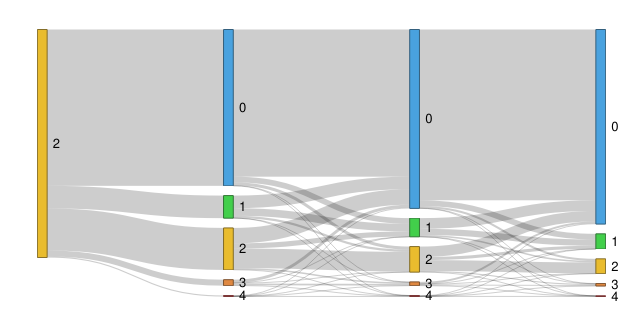


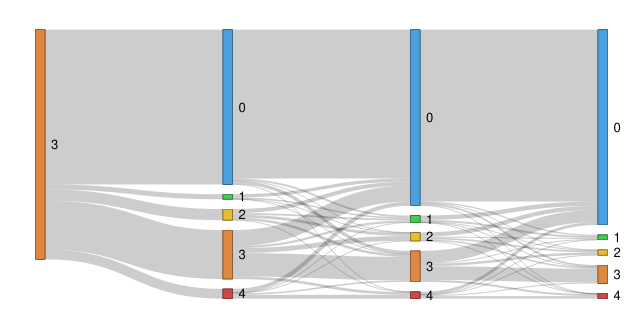

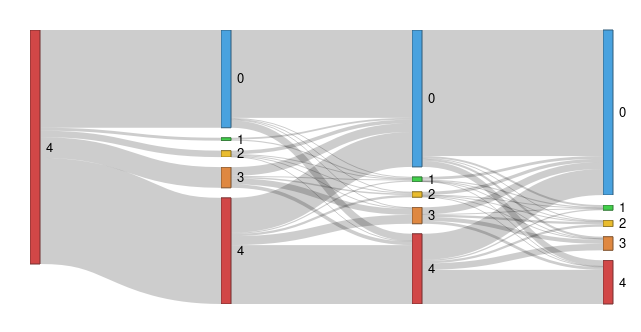


Each vertical bar denotes the index date, 6-months, 12-months and 24-months after index date. 0: off treatment; 1: <50 MME/day; 2: 50-119 MME/day; 3: 120-199 MME/day; 4: ≥200 MME/day.

**Daily MME transition over 6 months, 1-year, 2, years**

| Starting dose  (First 3 months) | Transition to | 6 months | 1 year | 2 year |
| --- | --- | --- | --- | --- |
| <50 (n= 1,925,944) | <50 | 330212 (17.15) | 266090 (13.82) | 212791 (11.05) |
|  | 50-119 | 5482 (0.28) | 5639 (0.29) | 5433 (0.28) |
|  | 120-199 | 434 (0.02) | 726 (0.04) | 849 (0.04) |
|  | ≥200 | 91 (0.00) | 222 (0.01) | 297 (0.02) |
|  | Withdrawn | 1589725 (82.54) | 1653267 (85.84) | 1706574 (88.61) |
| 50-119 (n= 24,315) | <50 | 2408 (9.90) | 1993 (8.20) | 1627 (6.69) |
|  | 50-119 | 4479 (18.42) | 2726 (11.21) | 1584 (6.51) |
|  | 120-199 | 641 (2.64) | 407 (1.67) | 278 (1.14) |
|  | ≥200 | 118 (0.49) | 116 (0.48) | 80 (0.33) |
|  | Withdrawn | 16669 (68.55) | 19073 (78.44) | 20746 (85.32) |
| 120-199 (n= 4,332) | <50 | 95 (2.19) | 134 (3.09) | 97 (2.24) |
|  | 50-119 | 210 (4.85) | 162 (3.74) | 105 (2.42) |
|  | 120-199 | 924 (21.33) | 585 (13.50) | 346 (7.99) |
|  | ≥200 | 184 (4.25) | 131 (3.02) | 101 (2.33) |
|  | Withdrawn | 2919 (67.38) | 3320 (76.64) | 3683 (85.02) |
| ≥200 (n= 1,446) | <50 | 18 (1.24) | 30 (2.07) | 32 (2.21) |
|  | 50-119 | 40 (2.77) | 34 (2.35) | 40 (2.77) |
|  | 120-199 | 128 (8.85) | 102 (7.05) | 84 (5.81) |
|  | ≥200 | 656 (45.37) | 434 (30.01) | 270 (18.67) |
|  | Withdrawn | 604 (41.77) | 846 (58.51) | 1020 (70.54) |
