# Supplementary material for: Time trends and prescribing patterns of opioid drugs in UK primary care patients with non-cancer pain: A retrospective cohort study
Source: PLoS Med. 2020 Oct 15;17(10):e1003270. doi: 10.1371/journal.pmed.1003270 (PMC7561110; doi:10.1371/journal.pmed.1003270)
Supplement: S4 Fig — (A) Regions. (B) Practices. (C) Prescribers. (DOCX) [file pmed.1003270.s005.docx]

**S4 Fig: Level of variation among regions (A), practices (B) and prescribers (C) in terms of the odds of long-term opioid use**

**
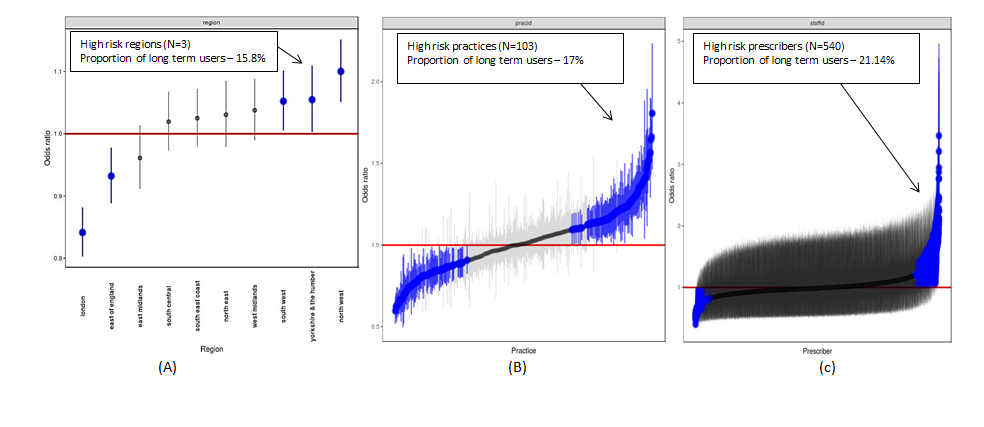
**
