# Supplementary material for: Time trends and prescribing patterns of opioid drugs in UK primary care patients with non-cancer pain: A retrospective cohort study
Source: PLoS Med. 2020 Oct 15;17(10):e1003270. doi: 10.1371/journal.pmed.1003270 (PMC7561110; doi:10.1371/journal.pmed.1003270)
Supplement: S5 Fig — (DOCX) [file pmed.1003270.s006.docx]

**S5 Fig: Risk of long-term opioid use for an individual in relation to prescriber and practice:**

**
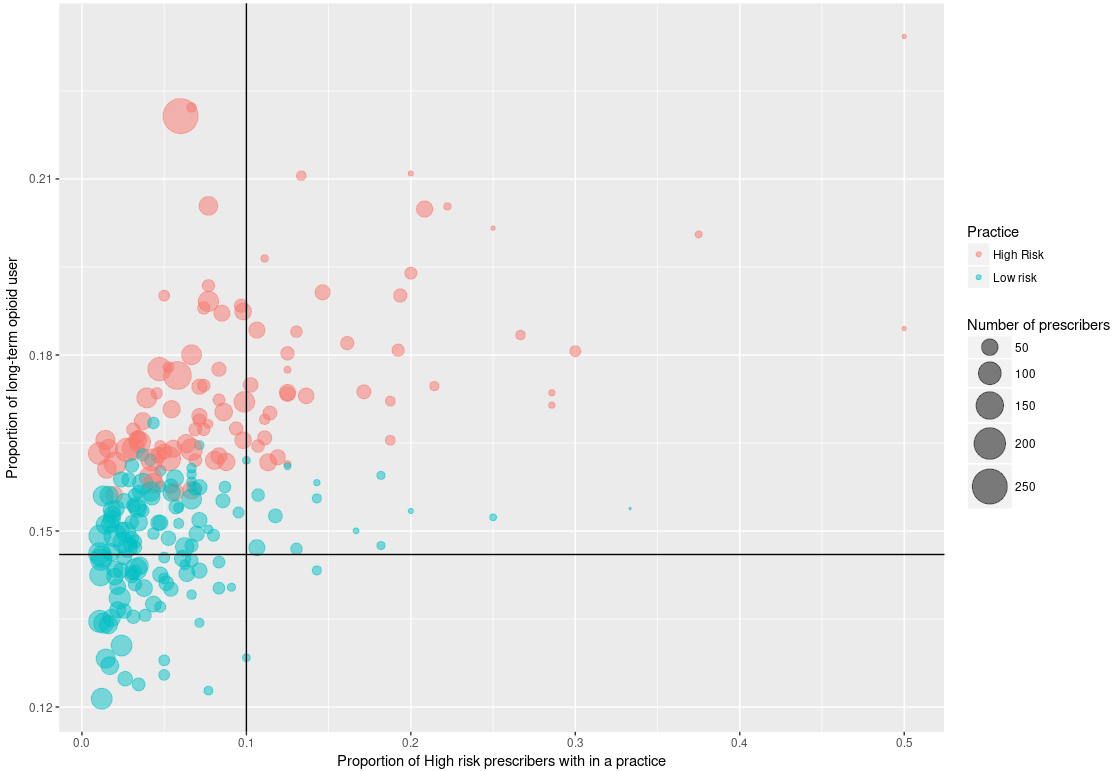
**

Practice A

The Y axis denotes the estimated proportion of long-term user for each practice after adjusting for case mix and x axis is the proportion of high risk prescribers with in each practice. The size of the bubbles represents the number of prescriber each practice have. For example, Practice A in the above plot have 20 prescribers and 6 (30%) of them are identified as high-risk prescribers and the estimated proportion of long-term users in this practice is 0.18. Most of the red bubbles were observed in the top right corner suggesting practices become a high risk due to small (<10%) high risk prescribers.
